# Supplementary material for: The construction of placeness in traditional opera from the perspective of structuration theory: A case study of Huangmei Opera in Anqing, China
Source: PLoS One. 2025 Oct 13;20(10):e0334133. doi: 10.1371/journal.pone.0334133 (PMC12517519; doi:10.1371/journal.pone.0334133)
Supplement: S1 File — (DOCX) [file pone.0334133.s001.docx]

# **Supporting information**

**S1 File.Interview outline**

Time：__________ Location：________ Status：_____________

Gender：__________Age：____________ Number：_____________ （ZF/CY/JM/WD-00）

**Interview subjects: Local government**

1. What activities related to the Huangmei Opera are organized by the local government of Anqing? (Regular activities, festivals/competitions, etc.)

2. What are the specific implementation plans for the various activities organized?

3. How do different groups participate? (Communities, scenic spots, campuses, theater troupes, etc.)

4. How effective are the various activities organized?

5. What guarantees and support policies does the government provide?

6. What are your suggestions for the localization of Huangmei Opera?

**Interview subjects: Huangmei Opera practitioners**

1. What is your connection to Huangmei Opera? How did you become involved with it?

2. What does Huangmei Opera mean to you? What has motivated you to continue dedicating yourself to the preservation and promotion of Huangmei Opera culture?

3. What do you consider to be the distinctive features of Huangmei Opera? Do you believe these features are connected to Anqing?

4. What are the formats of the troupe’s performance activities? (e.g., charitable performances, commercial performances, etc.)

5. What factors are generally considered when selecting themes and content for new plays?

6. How long does it take for a significant production to go from creation to final presentation? Could you share the process?

7. How do you view the reform and innovation of Huangmei Opera? In the process of reform and innovation, what do you think should be preserved, and what can be innovated?

8. Please discuss your perspective on the current state of the opera environment. For example, what is the level of government support, and how do changes in the audience market affect it?

**Interview subjects: Residents**

1. Do you enjoy listening to Huangmei opera? If so, do you go to the theater, or do you listen to it in places like parks and squares? Do you choose to watch videos or listen to audio recordings to enjoy Huangmei opera?

2. Please talk about your knowledge or memories of Huangmei opera. For example, what was Huangmei opera like when you were a child? Is it different from Huangmei opera today? Do many of your family members or friends enjoy Huangmei opera?

3. Do you frequently encounter Huangmei Opera in your daily life? For example, where?

4. Do you sing Huangmei Opera yourself? Have you ever considered teaching your family this skill? For example, would you be willing to have your children learn Huangmei Opera?

5. How do you view the connection between Huangmei Opera and the Anqing region?

6. When you listen to Huangmei Opera, does it remind you of any particular events or places?

7. In your opinion, what is the most critical element for the development and preservation of Huangmei Opera?

8. If your friends were to visit Anqing, would you choose to take them to watch a performance of Huangmei Opera?

**Interview subjects: Cultural consumers**

1. Where are you from? What methods do you typically use to enjoy Huangmei Opera in your daily life?

2. When listening to the Huangmei Opera, what aspects do you pay particular attention to?

3. Why do you focus on these aspects? What do they mean to you?

4. Could you share your understanding or opinions about Huangmei Opera? For example, its origins and development.

5. How do you view the connection between Huangmei Opera and Anqing?

6. When listening to the Huangmei Opera, do you associate it with any particular events or places?

7. How do you view traditional plays versus newly adapted plays?

8. What suggestions do you have for the future development and preservation of Huangmei Opera?
