# Supplementary material for: The construction of placeness in traditional opera from the perspective of structuration theory: A case study of Huangmei Opera in Anqing, China
Source: PLoS One. 2025 Oct 13;20(10):e0334133. doi: 10.1371/journal.pone.0334133 (PMC12517519; doi:10.1371/journal.pone.0334133)
Supplement: S1 Table — (DOCX) [file pone.0334133.s002.docx]

# **Supporting information**

**S1 Table.General situation of professional troupe in Anqing**

| Name | District/county | Established/year | Type of unit |
| --- | --- | --- | --- |
| Anqing Huangmei Opera Art Theater | Jingkai district | 2010 | National ownership |
| Anhui Zaifen Huangmei Opera Culture and Art Co. | Yingjiang district | 2012 | Other joint stock companies |
| Qianshan Huangmei Opera Troupe | Qianshan city | 2010 | National ownership |
| Taihu County Huangmei Opera Performing Arts Co. | Taihu county | 2010 | Limited liability company (wholly state-owned) |
| Huaining County Huangmei Opera Troupe | Huaining county | 2010 | National ownership |
| Tongcheng Huangmei Opera Art Communication Co. | Tongcheng city | 2004 | Limited liability companies (wholly owned by legal persons not invested or controlled by natural persons) |
| Wangjiang County Huangmei Opera Art Communication Co. | Wangjiang county | 2010 | Limited liability company (wholly state-owned) |
| Su Song County New Huangmei Opera Performing Arts Limited Liability Company | Susong county | 2010 | Limited liability company (wholly state-owned) |
| Yuexi Gaoqiang Heritage Center | Yuexi County | 2010 | business unit |
