# Supplementary material for: The construction of placeness in traditional opera from the perspective of structuration theory: A case study of Huangmei Opera in Anqing, China
Source: PLoS One. 2025 Oct 13;20(10):e0334133. doi: 10.1371/journal.pone.0334133 (PMC12517519; doi:10.1371/journal.pone.0334133)
Supplement: Attachment — Submitted filename: Response to Reviewers.doc [file pone.0334133.s004.doc]

Dear Editors and Reviewers，

We are very grateful to the reviewers for their valuable comments on our manuscript titled **"The Construction of Placeness in Traditional Opera from the Perspective of Structuration Theory: A Case Study of Huangmei Opera in Anqing, China" (ID: PONE-D-25-06193).** We have revised the manuscript in response to the reviewers' comments, questions, and suggestions.

We have made three major revisions to the paper. First, the introduction section has been significantly revised to highlight the innovative aspects of this specific method and strengthen the methodological argumentation. Second, the discussion section has been significantly revised to provide a distinct section separate from the conclusion. Third, all references have been verified and revised.

Additionally, we have attached the revised funding statement in the cover letter. We confirm that this study was supported by the Anhui Province Key Project for Scientific Research in Higher Education Institutions (2023AH050461), awarded to FZ; the Anhui Province Social Sciences Innovation and Development Research Project (2025CXZ015), awarded to FZ; and the Anhui Province Higher Education Institution Key Research Project (2024AH030073), awarded to LZ. This study did not receive any additional external funding support.

Regarding the data availability statement, We recognize the importance of data sharing in ensuring research transparency and reproducibility. However, given the sensitivity of the data in this study and our ethical commitment to research participants, we have chosen to restrict data sharing in this case. The raw data from this study includes qualitative interviews, which, even after anonymization, may still contain personal narratives that could compromise participants' privacy. By the ethical protocol approved by the Ethics Committee, we guarantee the anonymity of participants and the confidentiality of the data. The contact information for the Ethics Committee at Anqing Normal University is provided below for submitting data requests. Contact information: keyanc@aqtc.edu.cn. Although we cannot share the original interview data, the methods and tools used ensure the reproducibility of the study. To this end, we have detailed the interview guidelines and thematic analysis of the interviews in the "Methodology and sample description." Additionally, we have provided a detailed interview outline in the supplementary information file and cited excerpts from the interview texts in the manuscript. This will provide other researchers with the necessary information to understand and, if needed, replicate our research methods.

The reviewers' comments are listed below in bold italics, with specific issues numbered. Our responses are presented in regular font, and any revisions or additions to the manuscript can be viewed in the "Revised Manuscript with Track Changes."

**Reviewer 1**

***1.Please follow the journal author instructions. It would be useful for the reader to follow it. In general, the paper needs better organization.***

**Response to Q1：**We sincerely appreciate the valuable suggestions provided by the reviewers. We fully accept the recommendations regarding the need to better adhere to the journal's author guidelines and to enhance the paper's organization. In the revised manuscript, we have strictly followed the journal's latest author guidelines, meticulously reviewing and revising all details, including formatting, citation standards, and figure and table requirements, to ensure full compliance with the journal's requirements. Additionally, we have conducted a thorough review and optimization of the paper's overall structure, logical flow, and paragraph coherence. This includes re-examining the focus of the introduction and discussion, the clarity of the methods description, the presentation of results, and the conciseness of the conclusions. Our goal is to significantly enhance the paper's readability, coherence, and argumentative strength, thereby aligning it more closely with journal standards and making it easier for readers to understand. The revised sections can be viewed in the "Revised Manuscript with Track Changes."

***2.In the introduction, it would strengthen the argument on the methodology if it would be clearly stated what is innovative about this specific methodological development.***

**Response to Q2:** We sincerely thank the reviewers for their valuable comments. We fully agree that it is important to clarify the innovative nature of the methodology in the introduction to strengthen the methodological argument. To this end, we have made the following additions and improvements to the introduction in the revised manuscript:

In the third paragraph, we have added an introduction to the three-dimensional interactive model of structured theory that we have adopted, clearly pointing out its creative transformation as a theoretical paradigm. It not only breaks through the familiar dichotomy between structure and action in opera research but also introduces a dynamic perspective of “place as a process,” providing a new cognitive framework for understanding the modern transformation of traditional opera.

In the fifth paragraph, we explicitly state that, based on constructivist epistemology, we adopt a process-oriented qualitative analysis approach aimed at capturing the dynamic relationships among the three dimensions. We emphasize that this qualitative research design not only closely aligns with Pred's theoretical focus on "process" and "situationality" but also transcends cultural appearances to provide a profound elucidation of the complex logic underlying the construction of placeness.

In the sixth paragraph, we provide a supplementary explanation of the paper's overall structure to help readers better understand the argumentative framework and content organization of this study.

***3. In line with the latter, also balance and articulation of the methodological approach needs to be improved so that the application can be actually demonstrative of the validity of the methodology.***

**Response to Q3:** We appreciate the reviewers' important suggestions regarding the need to improve the balance and coherence of the methodology to demonstrate its validity effectively. We deeply understand the critical role that consistency and mutual support among the theoretical perspective, data manipulation, and analytical techniques play in demonstrating the rigor and validity of the research. To this end, in addition to strengthening the description of methodological innovation in the introduction, we have made the following key revisions in the "Methodology and sample description" of the revised manuscript:

(1)In the research design: The research value of Pred's structured theoretical perspective is explained in detail and closely combined with the subject and specific research content of this study, the applicability of this methodology in solving the research problems, and the design ideas of its analytical framework are systematically discussed to ensure that the theoretical framework is closely linked to the empirical analysis objectives.

(2) In the data source: The description has been enhanced to present the specific methods and sources of data collection, as well as the core content, more clearly and systematically, thereby improving the transparency and operability of the data acquisition process.

(3) Supplementary data analysis: This section provides a detailed introduction to the inductive coding process used in this study, explaining the specific steps involved in gradually forming, refining, and validating themes from the raw data, thereby ensuring the traceability of the analysis process.

(4) Supplementary ethical considerations: Clearly state that the study has been reviewed and approved by the Research Ethics Committee of Anqing Normal University and that all research procedures comply with the principle of informed consent. Participants are fully informed of the study's purpose, the use of their data, and the strict anonymization measures in place to ensure the standardization and ethical nature of the research process.

***4.Improve the title.Use different keywords from title. Improve the keywords.***

**Response to Q4:** We want to express our gratitude to the reviewers for their constructive suggestions regarding the improvement of the title and keywords. We fully recognize that a clear, accurate, and informative title structure, along with appropriate keywords, is crucial for enhancing the discoverability, readability, and overall structural clarity of the paper. In response to this feedback, we have systematically optimized the title hierarchy and keywords in the revised manuscript:

(1)Clarification of the overall structure: We reorganized and standardized the first-level headings, clearly setting them as Introduction, Literature Review, Methodology and sample description, Results, Discussion, Conclusions, and Limitations of the study. This ensures that the overall framework of the paper adheres to academic norms and is logically coherent.

(2) Refined chapter content: The literature review includes "Placeness formation mechanisms" and "Relationship between text and placeness," focusing more precisely on core theoretical issues. The methodology and sample description include "Study design," "Data sources," "Data analysis," and "Ethical considerations," significantly enhancing the transparency, clarity, and practicality of the methods section. The results include Structural elements: cultural system reform, Actor practice: Subjects of action and their practical responses, Elements of placeness: foundations and constructs, and Role mechanisms of Huangmei Opera's participation in the construction of placeness. These subheadings succinctly summarize the core findings and highlight the three-dimensional analytical framework of the structured theory. The Discussion includes Intertwined dynamics, breaking through the "structure-action" dichotomy, Consultations on modernity, placeness production of "sharing" and "progress," and the Path of living heritage, balancing place roots and global perspective. These titles not only distill the core arguments but also effectively link theoretical breakthroughs, reflections on modernity, and heritage protection pathways, demonstrating the depth and breadth of the Discussion.

(3) Keyword optimization: In conjunction with revising chapter titles, we carefully evaluated and improved keywords to ensure that they more comprehensively and accurately reflect the paper's core research subjects, theoretical perspectives, core concepts, and research contributions.

***5.I propose to the authors to be more specific, explanatory and simplified in order to be easily understandable from the readers.***

**Response to Q5:** We sincerely thank the reviewers for their valuable suggestions, which emphasize the need for greater specificity and conciseness in explanations to enhance reader comprehension. We fully agree that clear and concise expression is crucial for academic communication. To address this feedback, we have systematically reviewed the entire text, with a particular focus on revising the “Results.” We have refined core points, removed redundant information, optimized sentence structure, and improved logical coherence to ensure that the presentation of research findings is more focused, direct, and easy to understand.

***6.Results and discussion is too short. Need the give exactly discussions section.***

**Response to Q6:** We are grateful to the reviewers for pointing out that the results and discussion were insufficient in length and needed more precise discussion. We fully understand the importance of elaborating on the results and engaging in in-depth theoretical dialogue in order to demonstrate the value of the research. To this end, in the revised manuscript, we have optimized the structure and expanded the content of these two sections.

(1)Results section: We merged the two sections previously titled "Driving factors of Huangmei Opera's participation in placeness construction" and "Mechanisms of action." This adjustment was based on the fact that both sections were directly derived from data analysis and were logically closely related. The driving factors form the foundation for the mechanisms to operate, while the mechanisms are the concrete manifestation of the driving factors. The merged section presents the core findings in a more focused and coherent manner, illustrating the interactive relationships within the three-dimensional framework of structured theory.

(2) Discussion section: This section has been significantly expanded and strengthened. We have dedicated an entire chapter to this topic, focusing on how this study overcomes the limitations of existing research by integrating structured theory with placeness and what new theoretical insights or analytical frameworks it contributes. We provide an in-depth explanation of the complex dynamic processes, key driving factors, and their interactive mechanisms, as revealed by the research findings regarding Huangmei Opera's participation in place identity construction, thereby deepening our understanding of the phenomenon's essence. Based on the research findings, we explore the practical significance and development pathways of this case study for achieving living heritage transmission of traditional opera in a contemporary context, reconstructing place identity, and balancing place roots with a global perspective.

***7.Results section need to make separate from discussion and give whole results for discussion part.***

**Response to Q7:** We sincerely thank the reviewers for their guidance in clearly distinguishing between the results and the discussion and for comprehensively explaining the value of the results within the discussion. We fully understand the necessity of clearly separating research findings from their theoretical significance and practical implications in order to enhance the rigor and depth of argumentation in the paper. To this end, we have made key structural adjustments and content enhancements in the revised manuscript:

(1)Structural separation: In strict accordance with the opinion, the “Conclusion” has been separated from the “Discussion” to ensure that each section is independent and has a clear function.

(2) In-depth discussion: In the independently structured "Discussion," we systematically and thoroughly elucidate the overall value and implications of the research findings, focusing on how this study breaks through the limitations of the traditional "structure-action" dichotomy and provides new theoretical frameworks or insights. Based on all research findings, we summarize and distill the core processes of Huangmei Opera's participation in placeness construction, particularly the generative logic and interactive mechanisms of the key driving forces of "sharing" and "progress," deeply revealing the dynamism and complexity of placeness production. We propose feasible pathways for the living inheritance of traditional opera based on research findings, emphasizing their practical significance in balancing traditional preservation and innovative development, maintaining local roots, and connecting with broader perspectives.

***8.Conclusion section is short and need to improve.***

**Response to Q8:** We are grateful to the reviewers for pointing out that the conclusion was insufficient in length and needed to be strengthened. We sincerely acknowledge that a concise, comprehensive, and insightful conclusion is essential for summarizing the research's value, highlighting its contributions, and indicating future directions. To this end, we have significantly expanded and deepened the conclusion in the revised manuscript:

(1)Systematic summary of core contributions: Distilled and articulated the core contributions of this study at the theoretical, process mechanism, and practical levels.

(2) Strengthening the argumentation of research findings: Based on the results and discussion, the key driving factors and mechanisms of Huangmei Opera's participation in placeness construction, as well as the "shared" and "progressive" placeness characteristics that ultimately resulted from this participation, are argued more fully and specifically, ensuring that the conclusions are rooted in solid research evidence.

(3) Fully extracting research value: The research value has been further elaborated, emphasizing that this case not only reveals the internal mechanisms of the living inheritance of traditional opera but also provides important theoretical insights and practical references for understanding the resilience, adaptability, and creative development of local cultures in the era of globalization.

***9.Correct references in the text and the reference list according to the journal's format.***

**Response to Q9:** We sincerely thank the reviewers for their valuable suggestions. In response to these comments, we have completed a comprehensive revision, carefully checking all literature entries to ensure that author names, titles, sources, DOI, and other elements fully comply with the latest requirements of PLOS ONE. At the same time, we have verified all references to ensure that the cited references are indeed relevant to the text.

**Reviewer 2**

***1.Use of technical jargon and conveyance of clear points for the reader should be balanced. This could improve communication of the research process and findings to field experts and non-experts or mere field practitioners.***

**Response to Q1:** We sincerely thank the reviewers for their valuable suggestions. In response to the suggestion to “balance professional terminology with clarity of ideas,” we have completed a systematic optimization, providing explanations for core concepts such as “structure-action-place” inner construction when they first appear, retaining key terms in theoretical deductions and using action-oriented language in practical recommendations. Throughout the text, we have further improved readability for readers while ensuring academic rigor.

***2.Use of standard English, as recommended by PLOS ONE, helps to observe word economy, clarity of the message, and text conciseness. This helps even where manuscript word limit is imposed. For example, a sentence such as, “Therefore, it is necessary to comprehensively consider the diversified subjects of the construction of placeness…”, can be rewritten as, “Therefore, it is necessary to comprehensively consider the diversified subjects of placeness construction…”. In this case, two words are eliminated but the message is conveyed in a concise manner.***

**Response to Q2:** We sincerely thank the reviewers for their precise guidance on language efficiency. We have followed PLOS ONE standards to systematically streamline the entire text, remove redundant phrases, strengthen the subject voice, and compress conjunctions to ensure conciseness.

***3.The last paragraph of the introduction could be enhanced, by explicitly stating the knowledge gap(s) in the literature. At the same time, to arouse the reader’s interest, the aim/objectives/hypotheses of the paper should be clear. Further, he paper’s main contribution to the literature could be highlighted, as a punchline for the paper’s significance. Then the rest of the paper tries to show how you arrived at you claimed outcomes.***

**Response to Q3:** We want to thank the reviewers for their valuable comments on the introduction section. We have revised the introduction section accordingly:

(1)Identifying knowledge gaps: Pointing out that existing research has primarily focused on “policy or market-driven factors” and lacks a systematic analysis of the “triadic interplay between structure, action, and place.” Detailed revisions are outlined in the third paragraph of the introduction.

(2) Strengthen research objectives: Focus on “process” and ‘context’ as objectives, and set “analyzing the inter-constructive paths of Anqing Huangmei Opera” as a specific objective. Detailed revisions are outlined in the fifth paragraph of the introduction.

(3) Key academic contribution: This study diverges from the unidirectional determinism perspective and applies Pred's structured theory to the field of traditional opera, offering a mechanistic explanatory framework for the living inheritance of traditional opera.

***4.Sections and subsections not numbered. As the manuscript currently stands, it is easy for the reader to lose sense of the section they are reading, because the main threads of the arguments in the manuscript sound similar in many sections. Numbering sections and subsections could help to enhance the logical flow of thoughts.***

**Response to Q4:** We would like to express our gratitude to the reviewers for their valuable suggestions regarding the paper's structure. We have revised the paper by the formatting requirements of PLOS ONE, using first-level, second-level, and third-level headings. In particular, in the results section, we have combined the driving factors and mechanisms of action. The revised results section now includes Structural elements: cultural system reform, subjects of action and their practical responses, Elements of placeness, and Role mechanisms of Huangmei Opera's participation in the construction of placeness. This is intended to facilitate readers' comprehension and understanding.

***5.First part of the Methodology section sounds like an extension of the Literature Review. Generally, sections should have their own distinct character, theme, aspect or angle of the research presentation. Even though most of the research process is well explained (e.g. data collection), a table could assist the reader to properly follow and contextualize the data sources, and link that to the kind of data they provided, whether it was for the structural, action or place aspects of the research in this case.***

**Response to Q5:** We sincerely thank the reviewers for their valuable comments on the methodology section. We fully agree with your view that the methodology chapter should have clear independence and a unique research perspective, avoiding confusion with the content of the literature review. Regarding your point that the first part resembles an extension of the literature review, we will carefully revise the content to ensure that the first part of the methodology section focuses on the actual analytical framework of this study, distinctly separating it from the theoretical foundations of the literature review, thereby highlighting the unique value of the methodology. Detailed revisions are outlined in “Study design.”

At the same time, we strongly agree with your suggestion to clarify the data sources. The table you proposed is an excellent solution. We have added a structured data source summary table to the revision, which systematically presents the sample composition of the interview subjects and details the data analysis methods and processes. For detailed revisions, please refer to “Data sources.”

***6.Figure locations indicated within sentences. Is it not possible to show these in separate lines bounded by parentheses or square brackets between paragraphs? For example, [Fig. 1 to be inserted here]. Is it not a requirement to state the Figure sources? i.e. whether copied or self-generated?***

**Response to Q6:** We appreciate the reviewers' detailed suggestions regarding the labeling of figures and tables. We will strictly follow this format during revision to ensure that all figures and tables are labeled independently in separate lines. Regarding the requirement to provide source information for figures and tables, we confirm that all figures and tables in the paper are original.

***7.First sentences in some paragraphs of the Results section appear to be subsection headlines. Otherwise they don’t seem to convey any message and they could be eliminated since they are redundant.***

**Response to Q7:** We appreciate the reviewers' careful review of the structure of the results. We fully agree with your opinion regarding the redundancy of the first sentences of the paragraphs. Upon review, these sentences did indeed tend to be like titles, providing little substantive information. In the revised version, we have systematically deleted all such redundant first sentences and reorganized the logic of the relevant paragraphs. The current version ensures that each paragraph directly addresses the core research findings at the outset, eliminating issues of repetitive wording. The academic rigor of the revised manuscript has been enhanced, and we appreciate your bringing this important issue to our attention.

***8.Not sure if the Huangmei Opera location description fits well in the Results section. Could this be placed in another, or in a completely new section?***

**Response to Q8:** We appreciate the reviewers' thoughtful suggestions regarding the placement of the description of Huangmei Opera placeness in the results. We believe that the core rationale for including this content in the results chapter is that these placeness characteristics are not merely geographical background information but rather key empirical findings distilled through a systematic analysis of local annals, historical archives, and play texts. They reveal how spatial elements function as cultural driving forces, specifically participating in the construction of placeness in Huangmei Opera. If these descriptions were moved to the introduction or methodology, it would disrupt the logical chain between "cultural dynamics" and "constructive outcomes." To better align with your suggestions, we have made two optimizations in the revision: (1) added a topic sentence at the beginning of the paragraph to explicitly state the causal relationship between location descriptions and placeness construction; (2) highlighted case evidence to emphasize the empirical foundation of the conclusions.

***9.The important theoretical/historical backgrounds presented in the Results section should be minimized, and probably transferred to the Literature Review section.***

**Response to Q9:** We sincerely thank the reviewers for pointing out the imbalance between theory and historical background in the results. We fully agree that the results should focus on core research findings. In the revision, we have strictly adhered to the principle of conciseness, transferring macro-level theoretical developments and basic historical contexts, compressing key background information that needed to be retained, and refining the presentation of conclusions.

***10.For avoidance of doubt, an acronym such as CCTV should spelt out in full first time. Otherwise it could make the reader wonder whether the Authors were referring to Closed Circuit Television or China Central Television.***

**Response to Q10:** We sincerely thank the reviewers for their important reminder regarding the standardization of professional terminology. We fully agree with the academic principle that abbreviations should be accompanied by their full names when they first appear. During the revision of the entire text, we systematically checked all professional abbreviations. We added the full names of terms that could cause ambiguity, such as “China Central Television,” when they first appeared in the text.

***11.Lastly, sentence structure and grammar should be improved upon throughout the manuscript, to ensure clarity of thought and better communication.***

**Response to Q11:** We sincerely thank the reviewers for their insightful comments on the language quality of the manuscript. We have reviewed the entire text and completed revisions, focusing on optimizing complex sentence structures to improve readability, correcting grammatical errors, and adding academic transition words to strengthen paragraph connections.

We would like to express our gratitude to the editors and reviewers for their valuable comments and suggestions. We have carefully revised the paper in response to your suggestions. All revisions in the revised draft are presented as annotations for your review. Please let us know if you require any further revisions.

Sincerely,

Fang Zhu
